# Supplementary material for: Elemental stoichiometry and insect chill tolerance: evolved and plastic changes in organismal Na+ and K+ content in Drosophila
Source: Biol Open. 2024 Dec 30;13(12):BIO060597. doi: 10.1242/bio.060597 (PMC11708776; doi:10.1242/bio.060597)
Supplement: Supplementary information [file biolopen-13-060597-s1.pdf]

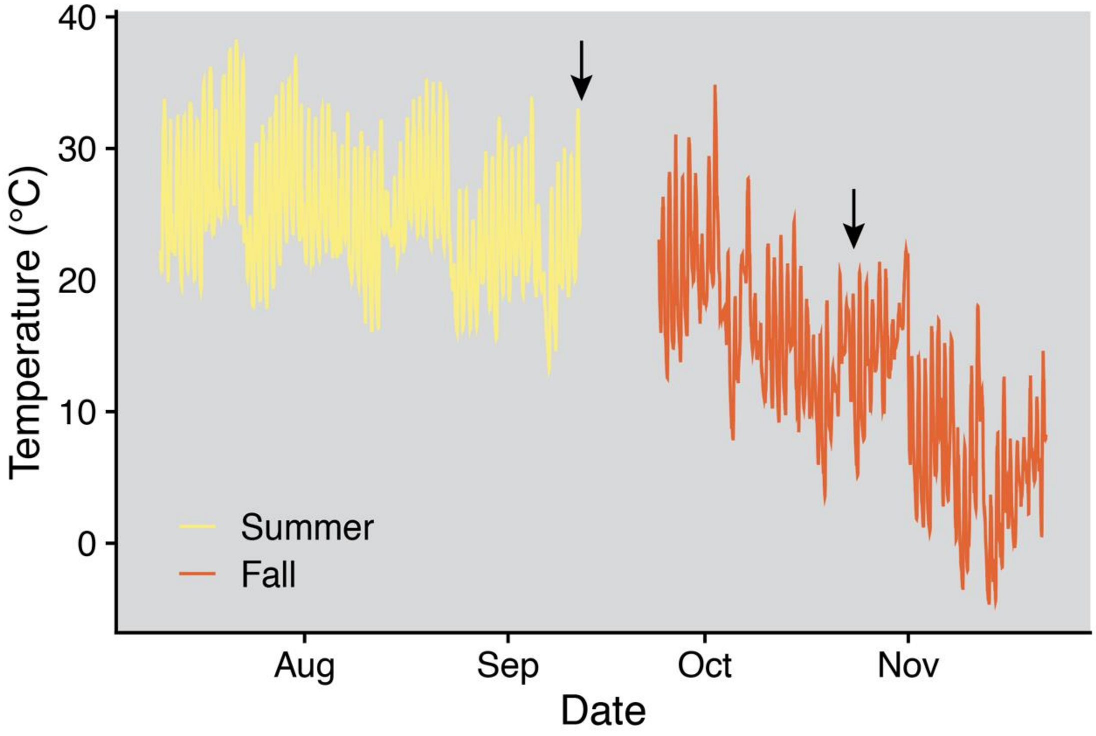

Fig. S1. Average hourly temperature from the seasonal adaptation experiment as measured by 8 independent temperature loggers. Arrows denote dates on which samples were collected.

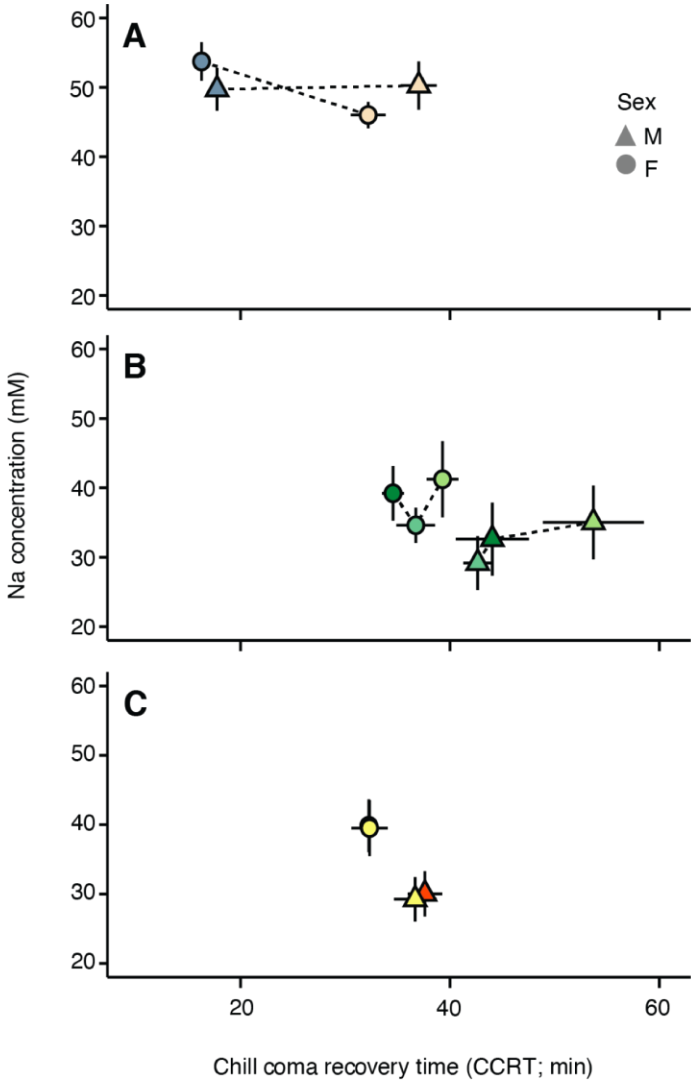

Fig. S2. Relationships between chill coma recovery time and average whole-body sodium concentration in both male (triangles) and female (circles) *D. melanogaster*. Means (± sem) among individuals following acclimation (A) or among lines collected at different locations (B) or collected in different seasons (C) are shown.

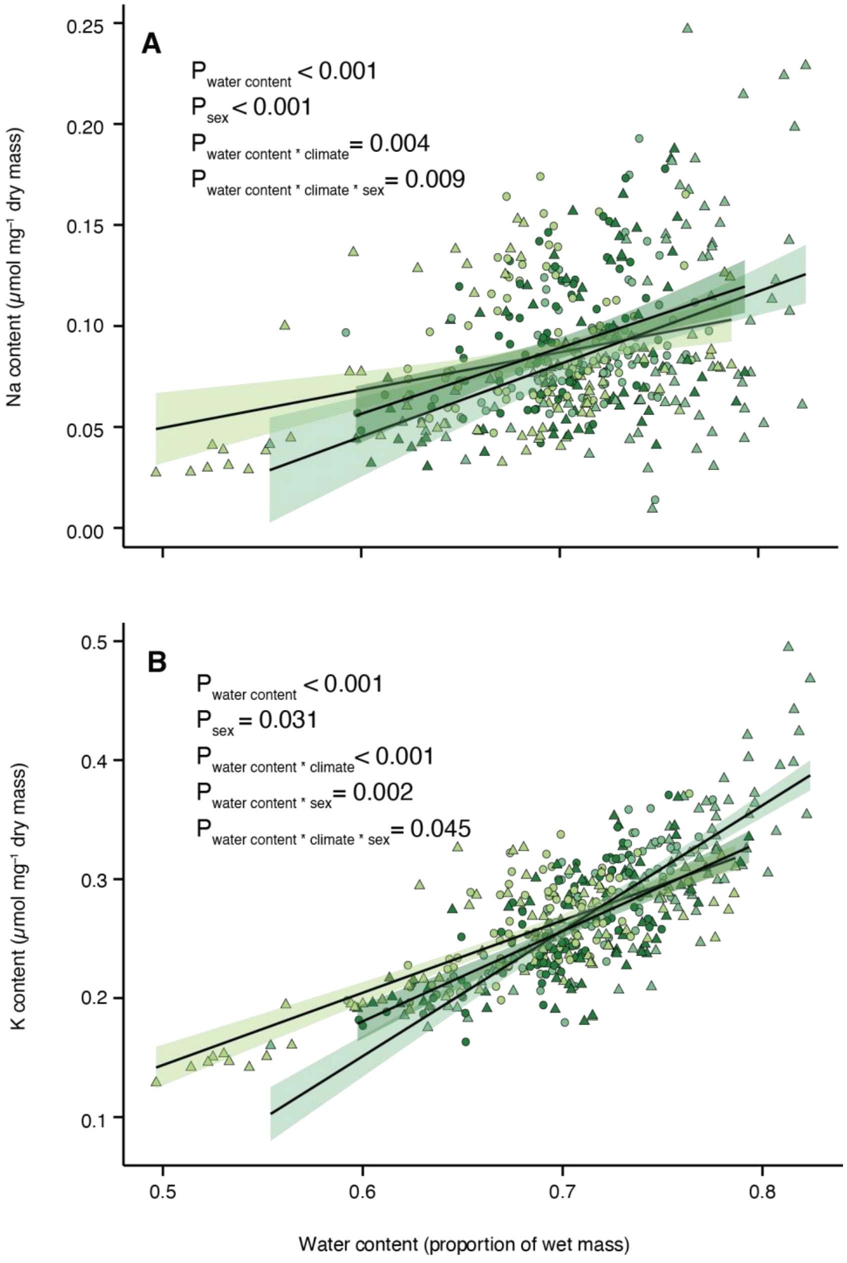

Fig. S3. Relationship between sodium or potassium content and water content in male (triangles) and female (circles) climate-adapted *D. melanogaster*. Solid black lines with transparent shaded area represent linear models to demonstrate trends (mean and sem of parameter estimates). Full results of mixed effects models are presented in Table S2.

Biology Open • Supplementary information

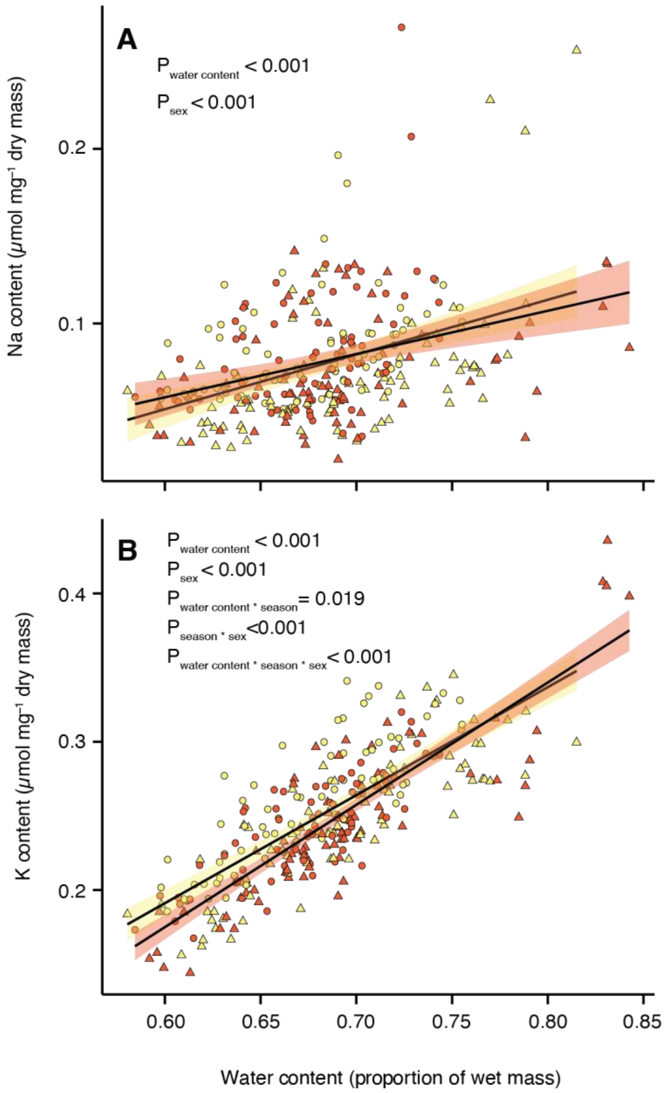

Fig. S4. Relationship between sodium or potassium content and water content in male (triangles) and female (circles) seasonally-adapted *D. melanogaster*. Solid black lines with transparent shaded area represent the overall data trend.

Biology Open • Supplementary information

Table S1. Summary of results from mixed-effects models on chill coma recovery time (CCRT). Effects of acclimation temperature, climate of collection, or season of collection and sex are shown for lines of *D. melanogaster* that have been acclimated, spatially adapted or temporally adapted (respectively). For flies collected from different climates or in different seasons, mixed-effects models included replicate line as a random effect. Shaded rows indicate significance based on *P*<0.05.

Group	Effect	F	*P*-value	df
Acclimation	Acclimation Temp.	178.52	< 0.0001	1, 106
Acclimation	Sex	7.38	0.008	1, 106
Acclimation	Acclimation Temp. * Sex	0.98	0.325	1, 106
Spatial	Climate	1.85	0.200	2, 12
Spatial	Sex	45.21	< 0.0001	1, 501
Spatial	Climate * Sex	3.12	0.045	2, 501
Temporal	Season	0.12	0.739	1, 8
Temporal	Sex	11.42	0.001	1, 340
Temporal	Season * Sex	0.39	0.534	1, 340

Biology Open • Supplementary information

Table S2. Results of mixed effects models of ion and water content (WC) in acclimated, climate-adapted, or seasonally-adapted fly lines. Shaded rows indicate significance based on *P*<0.05.

Variable	Group	Effect	F	*P*-value	df
Na	Acclimation	Temperature	0.01	0.922	1, 94
Na	Acclimation	Sex	0.42	0.517	1, 94
Na	Acclimation	Temperature * Sex	1.65	0.203	1, 94
Na	Spatial	Climate	0.03	0.969	2, 12
Na	Spatial	Sex	6.59	0.011	1, 408
Na	Spatial	Climate * Sex	13.85	<0.0001	2, 408
Na	Temporal	Season	<0.01	0.980	1, 8
Na	Temporal	Sex	17.21	<0.0001	1, 276
Na	Temporal	Season * Sex	<0.01	0.999	1, 276
K	Acclimation	Temperature	34.63	<0.0001	1, 94
K	Acclimation	Sex	0.50	0.482	1, 94
K	Acclimation	Temperature * Sex	0.53	0.468	1, 94
K	Spatial	Climate	1.15	0.348	2, 12
K	Spatial	Sex	2.02	0.156	1, 408
K	Spatial	Climate * Sex	18.14	< 0.0001	2, 408
K	Temporal	Season	0.08	0.780	1, 8
K	Temporal	Sex	2.40	0.123	1, 276
K	Temporal	Season * Sex	6.78	0.010	1, 276
WC	Acclimation	Temperature	2.13	0.148	1, 94
WC	Acclimation	Sex	2.44	0.122	1, 94
WC	Acclimation	Temperature * Sex	0.07	0.790	1, 94
WC	Spatial	Climate	1.99	0.180	2, 12
WC	Spatial	Sex	12.86	0.0004	1, 408
WC	Spatial	Climate * Sex	24.00	<0.0001	2, 408
WC	Temporal	Season	<0.001	0.991	1, 8
WC	Temporal	Sex	34.17	<0.0001	1, 276
WC	Temporal	Season * Sex	0.01	0.922	1, 276

Biology Open • Supplementary information

Table S3. Results of generalized linear models and mixed effects models of ion content relative to water content (WC) in acclimated, climate-adapted, or seasonally-adapted fly lines. Shaded rows indicate significance based on *P*<0.05. Na:K = Ratio of Na relative to K in body.

Variable	Group	Sex	Effect	F	*P*-value	df
Na	Acclimation	Female	WC	8.59	0.005	1, 49
Na	Acclimation	Female	Temperature	2.99	0.090	1, 48
Na	Acclimation	Female	Temperature * WC	20.75	<0.001	1, 47
Na	Acclimation	Male	WC	3.11	0.084	1,47
Na	Acclimation	Male	Temperature	0.12	0.728	1, 46
Na	Acclimation	Male	Temperature * WC	3.65	0.062	1, 45
K	Acclimation	Female	WC	18.68	<0.001	1,49
K	Acclimation	Female	Temperature	31.99	<0.001	1, 48
K	Acclimation	Female	Temperature * WC	33.67	<0.001	1, 47
K	Acclimation	Male	WC	82.69	<0.001	1,47
K	Acclimation	Male	Temperature	27.14	<0.001	1, 46
K	Acclimation	Male	Temperature * WC	8.39	0.006	1, 45
Na:K	Acclimation	Female	WC	1.30	0.260	1,49
Na:K	Acclimation	Female	Temperature	0.163	0.163	1, 48
Na:K	Acclimation	Female	Temperature * WC	50.70	<0.001	1, 47
Na:K	Acclimation	Male	WC	1.49	0.229	1,47
Na:K	Acclimation	Male	Temperature	3.43	0.071	1, 46
Na:K	Acclimation	Male	Temperature * WC	2.70	0.107	1, 45

Biology Open • Supplementary information

Table S4. Results of generalized linear models and mixed effects models of ion content relative to water content (WC) in climate-adapted (locational), and seasonally-adapted fly lines. Shaded rows indicate significance based on $P<0.05$.

Variable	Group	Effect	F	*P*-value	df
Na	Locational	WC	60.33	<0.001	1, 411
Na	Locational	Climate	0.13	0.880	2, 12
Na	Locational	Sex	15.44	<0.001	1, 411
Na	Locational	WC * Climate	5.59	0.004	2, 411
Na	Locational	WC * Sex	0.07	0.796	1, 411
Na	Locational	Climate * Sex	2.48	0.085	2, 411
Na	Locational	WC * Climate * Sex	4.78	0.009	2, 411
K	Locational	WC	579.52	<0.001	1, 411
K	Locational	Climate	0.59	0.570	2, 12
K	Locational	Sex	4.70	0.031	1, 411
K	Locational	WC * Climate	25.68	<0.001	2, 411
K	Locational	WC * Sex	9.72	0.002	1, 411
K	Locational	Climate * Sex	0.61	0.611	2, 411
K	Locational	WC * Climate * Sex	3.13	0.045	2, 411
Na	Seasonal	WC	21.23	<0.001	1, 278
Na	Seasonal	Season	0.01	0.908	1, 8
Na	Seasonal	Sex	30.15	<0.001	1, 278
Na	Seasonal	WC * Season	0.28	0.599	1, 278
Na	Seasonal	WC * Sex	0.08	0.784	1, 278

Biology Open • Supplementary information

Na	Seasonal	Season * Sex	0.02	0.883	1, 278
Na	Seasonal	WC * Season * Sex	3.31	0.070	1, 278
K	Seasonal	WC	479.39	<0.001	1, 278
K	Seasonal	Season	0.44	0.528	1, 8
K	Seasonal	Sex	87.68	<0.001	1, 278
K	Seasonal	WC * Season	5.55	0.019	1, 278
K	Seasonal	WC * Sex	0.07	0.797	1, 278
K	Seasonal	Season * Sex	12.90	<0.001	1, 278
K	Seasonal	WC * Season * Sex	10.48	0.001	1, 278

Dataset 1.

Available for download at
https://journals.biologists.com/bio/article-lookup/doi/10.1242/bio.060597#supplementary-data
